# Cellular recovery from exposure to sub-optimal concentrations of AB toxins that inhibit protein synthesis

**DOI:** 10.1038/s41598-018-20861-9

**Published:** 2018-02-06

**Authors:** Patrick Cherubin, Beatriz Quiñones, Ken Teter

**Affiliations:** 1 Burnett School of Biomedical Sciences, College of Medicine, University of Central Florida, Orlando, Florida 32826 USA; 2 United States Department of Agriculture - Agricultural Research Service, Western Regional Research Center, Produce Safety and Microbiology Research Unit, Albany, California, 94710 USA

## Abstract

Ricin, Shiga toxin, exotoxin A, and diphtheria toxin are AB-type protein toxins that act within the host cytosol and kill the host cell through pathways involving the inhibition of protein synthesis. It is thought that a single molecule of cytosolic toxin is sufficient to kill the host cell. Intoxication is therefore viewed as an irreversible process. Using flow cytometry and a fluorescent reporter system to monitor protein synthesis, we show a single molecule of cytosolic toxin is not sufficient for complete inhibition of protein synthesis or cell death. Furthermore, cells can recover from intoxication: cells with a partial loss of protein synthesis will, upon removal of the toxin, increase the level of protein production and survive the toxin challenge. Thus, in contrast to the prevailing model, ongoing toxin delivery to the cytosol appears to be required for the death of cells exposed to sub-optimal toxin concentrations.

## Introduction

AB-type protein toxins are released into the extracellular environment but attack targets within the host cytoplasm^1,2^. These toxins initially enter the cell through receptor-mediated endocytosis and reach the cytosol between 30 min and 2 h after internalization from the plasma membrane^3–6^. Some AB toxins move into the cytosol from acidified endosomes, while others follow an inefficient transport pathway to the endoplasmic reticulum (ER) before entering the cytosol. Diphtheria toxin (Dtx) inhibits protein synthesis and belongs to the subset of toxins that cross the endosomal membrane to reach the cytosol^7^; ER-translocating toxins that inhibit protein synthesis include ricin, Shiga toxin 1 (Stx1), and exotoxin A (EtxA)^8–10^. Based on extrapolations from *in vitro* studies with toxin serial dilutions or kinetic analyses of intoxication^5,11,12^, it is believed the inhibition of protein synthesis and resulting cell death can result from the delivery of a single toxin molecule to the cytosol^5,11–16^. Dose response curves generated with AB toxins would thus reflect the probability of intoxication in a population of cells. By this model, which represents the current working paradigm, the half-maximal effective dose (ED_50_) of a toxin represents an all-or-nothing condition in which half the exposed cells contain no cytosolic toxin and are therefore unaffected while the other half exhibit the full effects of intoxication. An alternative interpretation for toxin ED_50_ values would be based on proportionality rather than probability: at the ED_50_ for protein synthesis inhibition, it is possible all cells in the exposed population contain an amount of cytosolic toxin that only reduces protein synthesis by 50%. With this proportionality model, limiting but not eliminating the quantity of cytosolic toxin could protect a cell from the lethal outcome of intoxication. This issue has important implications for inhibitor development, as the potentially lethal effect resulting from a single molecule of cytosolic toxin would greatly limit treatment regimes that are not 100% effective or target the cell-associated toxin after first contact. Here, we present data in support of the proportionality model that indicate ongoing toxin delivery to the cytosol is required for the death of cells exposed to sub-optimal toxin concentrations. Our work presents the first evidence with quantifiable data to challenge the “single molecule” paradigm of intoxication.

## Results

Most quantitative assays that monitor the toxin-induced inhibition of protein synthesis average the results from the entire population of cells^17,18^. This makes it difficult to distinguish between the “probability” and “proportionality” models of intoxication. As an alternative approach, we used flow cytometry in conjunction with a Vero cell line that expresses a destabilized variant of the enhanced green fluorescent protein (d2EGFP) that is degraded by the proteasome with a 2 h half-life^19^. This allowed us to record the toxin-induced inhibition of protein synthesis and resulting loss of EGFP fluorescence from individual cells. We have previously reported a direct, proportional link between the specific loss of d2EGFP fluorescence and the overall loss of protein synthesis in a population of toxin-treated cells^20^. Subsequent studies further documented the usefulness of the Vero-d2EGFP cells for measuring the activity of AB toxins^17,21–24^. Other investigators have used similar cell-based toxicity assays with destabilized reporters^25–27^ and have documented a direct correlation between the toxin-induced inhibition of total protein synthesis and the toxin-induced loss of reporter signal^28^.

**Figure 1:**
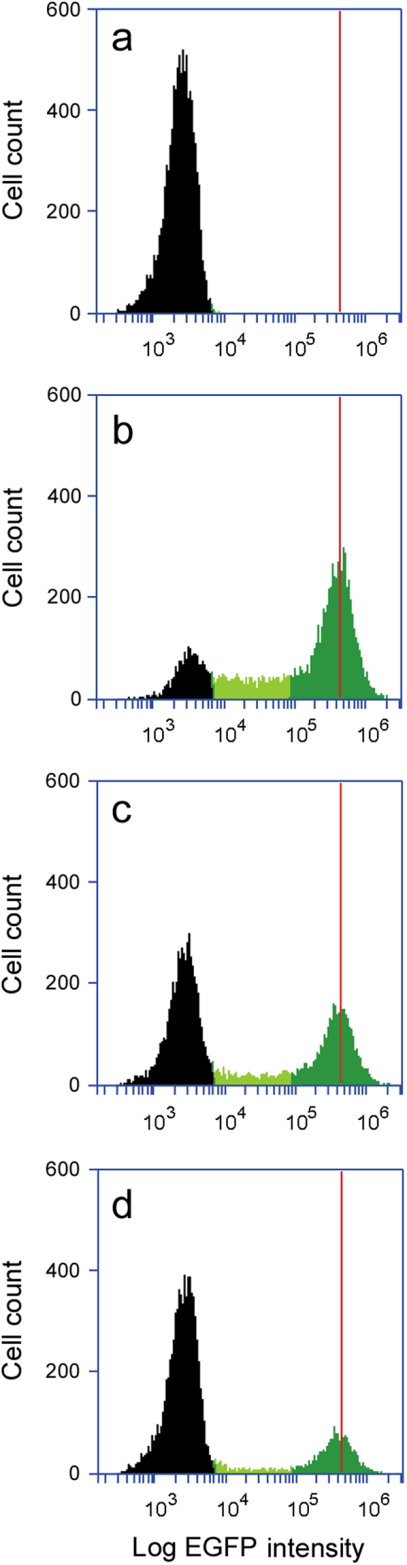
Detection of separate cell populations with or without EGFP expression. (**a**) Vero cells, (**b**) Vero-d2EGFP cells, (**c**) a 1:1 ratio of Vero:Vero-d2EGFP cells, and (**d**) a 4:1 ratio of Vero:Vero-d2EGFP cells were subjected to cytofluorometry. The range of background fluorescence generated by parental Vero cells and a minor population of the Vero-d2EGFP cells is in black. The distribution of higher levels of fluorescence for the Vero-d2EGFP cells is in dark green, while light green highlights the lower level of fluorescence from a subpopulation of Vero-d2EGFP cells. The peak fluorescent intensity from the population of Vero-d2EGFP cells with the highest fluorescence levels in panel b is represented by the red line in all panels.

Distinct populations of Vero and Vero-d2EGFP cells were resolved by cytofluorometry when the two cell types were mixed together (Fig. 1). The individual peaks of background autofluorescence (Fig. 1a) and EGFP fluorescence (Fig. 1b) from pure populations of Vero and Vero-d2EGFP cells, respectively, were both seen in mixed populations containing 1:1 (Fig. 1c) and 4:1 (Fig. 1d) ratios of Vero:Vero-d2EGFP cells. Although the number of cells contributing to the EGFP signal was reduced in the mixed populations, the peak fluorescent intensity from Vero-d2EGFP cells did not change. These results demonstrated it was possible to differentiate between populations of cells with or without EGFP expression.

**Figure 2:**
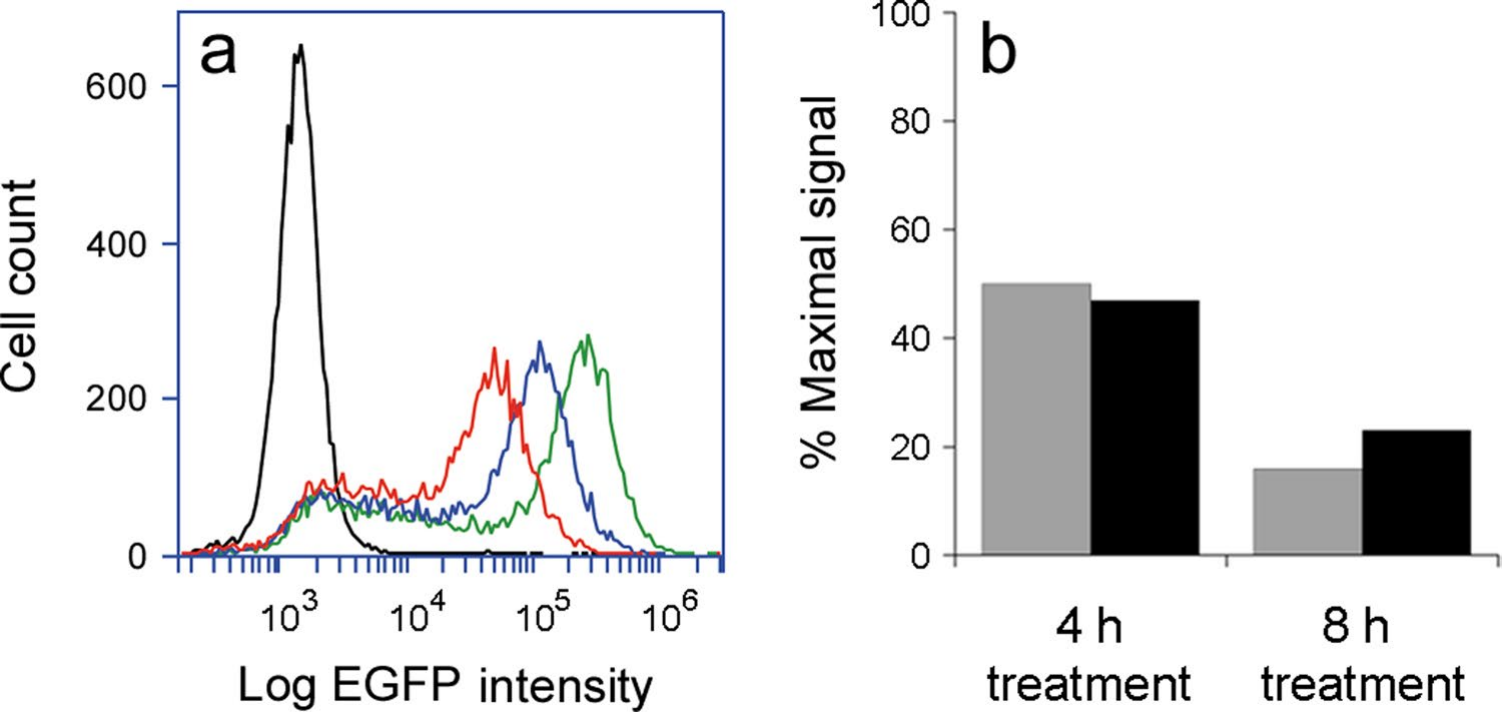
Population-wide loss of fluorescence from cycloheximide-treated Vero-d2EGFP cells. (**a**) Untreated parental Vero cells (black), untreated Vero-d2EGFP cells (green), or Vero-d2EGFP cells treated with cycloheximide for 4 h (blue) or 8 h (red) were subjected to cytofluorometry. (**b**) Using data collected from the same cells by either cytofluorometry (grey bars) or with a plate reader (black bars), signals from the cycloheximide-treated cells were expressed as percentages of the value recorded for untreated Vero-d2EGFP cells.

Consistent with previous reports^25,29^ a time-dependent reduction in the fluorescent intensity from cycloheximide-treated Vero-d2EGFP cells was detected by cytofluorometry and with a plate reader (Fig. 2). The fluorescent peak from untreated Vero-d2EGFP cells shifted to uniform peaks at progressively lower intensities after 4 h and 8 h incubations with cycloheximide, a protein synthesis inhibitor (Fig. 2a). Quantification of the remaining signals with both a plate reader and cytofluorometer recorded a 50% loss of fluorescence after 4 h of cycloheximide treatment and an ~80% loss of fluorescence after 8 h of cycloheximide treatment (Fig. 2b). Using an MTS assay, only a minor loss of cell viability (22%) was detected after an 8 h exposure to cycloheximide (*n* = 2, range = 3%). These pilot studies demonstrated it was possible to detect and quantify the population-wide loss of EGFP fluorescence resulting from exposure to an inhibitor of protein synthesis.

**Figure 3:**
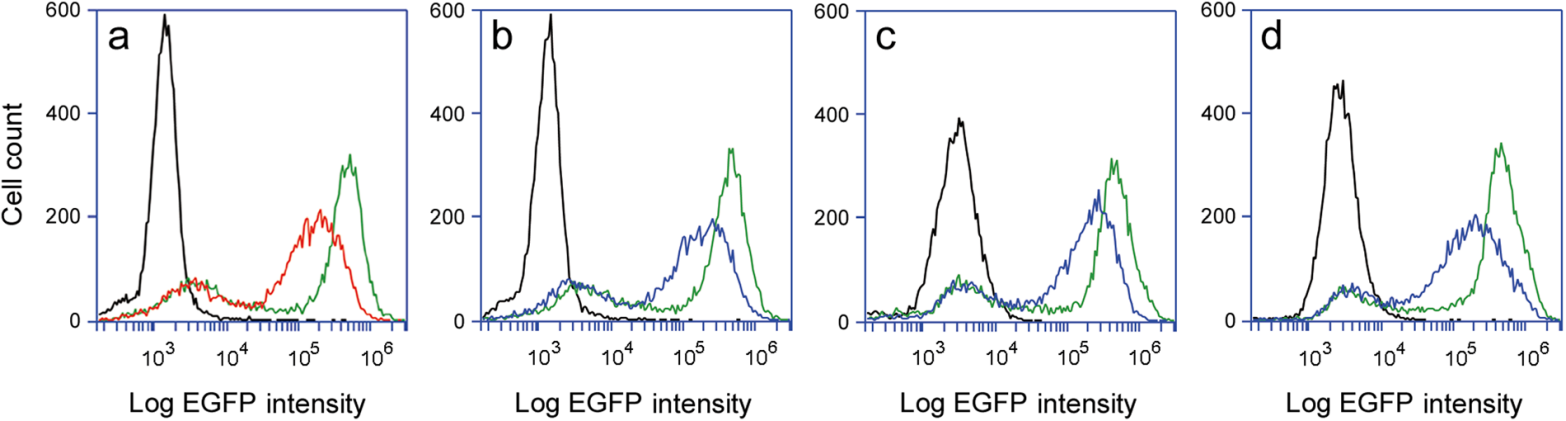
Population-wide loss of fluorescence from toxin-treated cells. Vero-d2EGFP cells (red or blue lines, corresponding to the color-coded toxin concentrations in Supplementary Figure S1) were subjected to cytofluorometry after a 20 h incubation with (**a**) 0.05 ng/mL of ricin, (**b**) 0.01 ng/mL of Stx1, (**c**) 1.0 ng/mL of EtxA, or (**d**) 0.05 ng/mL of Dtx. Unintoxicated parental Vero cells (black lines) and unintoxicated Vero-d2EGFP cells (green lines) were also processed for each condition.

We next used cytofluorometry to examine how EGFP fluorescence was affected in Vero-d2EGFP cells challenged with the AB toxins ricin (Fig. 3a), Stx1 (Fig. 3b), EtxA (Fig. 3c), or Dtx (Fig. 3d). In each case, cells were incubated with a toxin concentration that produced a roughly 50% reduction in EGFP fluorescence after 20 h of incubation. Because the fluorescence intensity is displayed on a log rather than linear scale, the cytofluorometry profiles corresponding to a 50% inhibition of protein synthesis were not observed exactly midway between the unintoxicated Vero-d2EGFP cells with maximal EGFP expression and the parental Vero cells without EGFP expression. If a single molecule of toxin could elicit a cytotoxic effect, then a 50% loss of EGFP fluorescence would represent a bimodal cell population: half the cells would be intoxicated with no protein synthesis, while the other half would be unintoxicated and therefore producing normal levels of protein with full EGFP fluorescence. However, our cytofluorometry data from intoxicated cells did not detect two distinct fluorescent peaks representing background fluorescence and a full EGFP signal. We instead recorded a uniform, population-wide downshift in mean fluorescent intensity of intoxicated Vero-d2EGFP cells. A bimodal distribution of cells with either full EGFP expression or no EGFP expression was clearly absent from the intoxicated cells. These results were similar to the effects observed in Vero-d2EGFP cells that had been treated with cycloheximide for 4 h and showed a population-wide downshift in their EGFP signal to 50% of the maximal value (Fig. 2). We accordingly concluded that the entire population of Vero-d2EGFP cells had been intoxicated, but the quantity of cell-associated toxin was only sufficient to reduce protein synthesis to 50% of normal levels.

A dose-dependent, population-wide loss of fluorescence was recorded for cells exposed to a range of concentrations for each of the tested AB toxins (Supplementary Fig. S1). This effect was confirmed by quantifying the fluorescent signal from intoxicated cells with a plate reader before collecting the cells for cytofluorometry. Furthermore, the loss of protein synthesis detected by cytofluorometry mirrored the loss of protein synthesis detected with a plate reader. Only cells treated with Stx1 or EtxA showed an obvious phenotypic effect from a 20 h challenge with higher toxin concentrations (Supplementary Fig. S2). MTS cell proliferation assays recorded a 25% loss of viability in cells treated with the highest concentration of Stx1 and less toxicity in cells challenged with lower Stx1 concentrations or any concentration of the other three toxins (Supplementary Fig. S2). For comparative purposes, the oxidative stress resulting from a 20 h exposure to 1 mM H_2_O_2_ lowered cell viability to 56 ± 3% (*n* = 3, ± std. dev.) of the untreated control value. These results indicated substantial cell death did not occur after a 20 h toxin challenge despite the reduction in protein synthesis.

**Figure 4:**
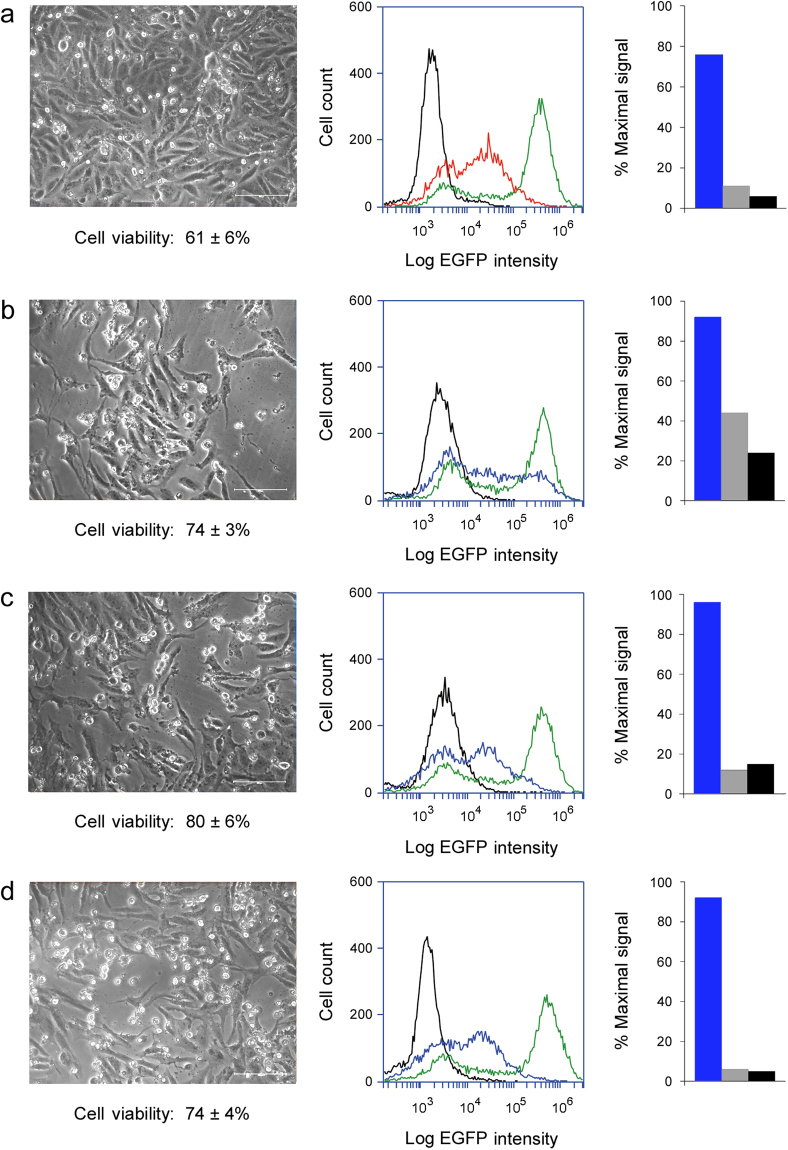
Cell survival with a long-term, toxin-induced inhibition of protein synthesis. Vero-d2EGFP cells were incubated for 36 h with (**a**) 0.05 ng/mL of ricin, (**b**) 0.01 ng/mL of Stx1, (**c**) 1.0 ng/mL of EtxA, or (**d**) 0.05 ng/mL of Dtx. Left column: Representative images were taken at 200 × magnification. Cell viability, as assessed by MTS assay (*n* = 3, avg. ± std. dev.), is indicated. Center column: Red and blue lines (corresponding to the color-coded toxin concentrations in Supplementary Figure S1) were generated from cytofluorometry analysis of the adherent subpopulation of toxin-treated Vero-d2EGFP cells. Unintoxicated parental Vero cells (black lines) and unintoxicated Vero-d2EGFP cells (green lines) were also processed for each condition. Right column: Cell viability was recorded by cytofluorometry analysis of annexin V and 7-AAD staining (blue), while EGFP fluorescence was recorded by cytofluorometry (grey) or with a plate reader (black). Results are presented as percentages of the values obtained from unintoxicated cells. All measurements in the matched center and right columns were performed on the same population of cells.

Our results demonstrated a single molecule of cytosolic toxin is not sufficient to completely inhibit protein synthesis and kill the target cell after a 20 h incubation. However, there is a lag between the loss of protein synthesis and cell death. We therefore extended our incubation with ricin (Fig. 4a), Stx1 (Fig. 4b), EtxA (Fig. 4c), or Dtx (Fig. 4d) to 36 h in order to examine whether extensive cell death follows the loss of protein synthesis. Some of the intoxicated cells had detached from the plate by 36 h, but cell viability (as assessed by MTS assay) was relatively high – between 60–80% of the unintoxicated control values (Fig. 4, left column). Cytofluorometry analysis of the remaining adherent cells documented a population-wide loss of protein synthesis (Fig. 4, center column). The fluorescent signals from these cells were substantially lower than the unintoxicated control values, yet most cells were still viable as demonstrated by a lack of staining with the apoptosis markers annexin V and 7-AAD (Fig. 4, right column). Viability as determined by MTS was lower than assessments made by annexin V / 7-AAD because the MTS assay accounted for the entire cell population whereas annexin V / 7-AAD staining only considered the subpopulation of adherent cells. Additional observations were recorded for a range of toxin concentrations, with the expected dose-dependent effects on cell morphology, viability, and protein synthesis (Supplementary Fig. S3). Surprisingly, however, almost none of the remaining adherent cells - even those with extremely low levels of protein synthesis - were apoptotic. A 36 h toxin challenge thus left a subpopulation of cells with a quantity of cytosolic toxin that inhibited protein synthesis but was not lethal. These observations strongly indicated that a single molecule of cytosolic toxin will neither completely inhibit protein synthesis nor induce cell death.

**Figure 5:**
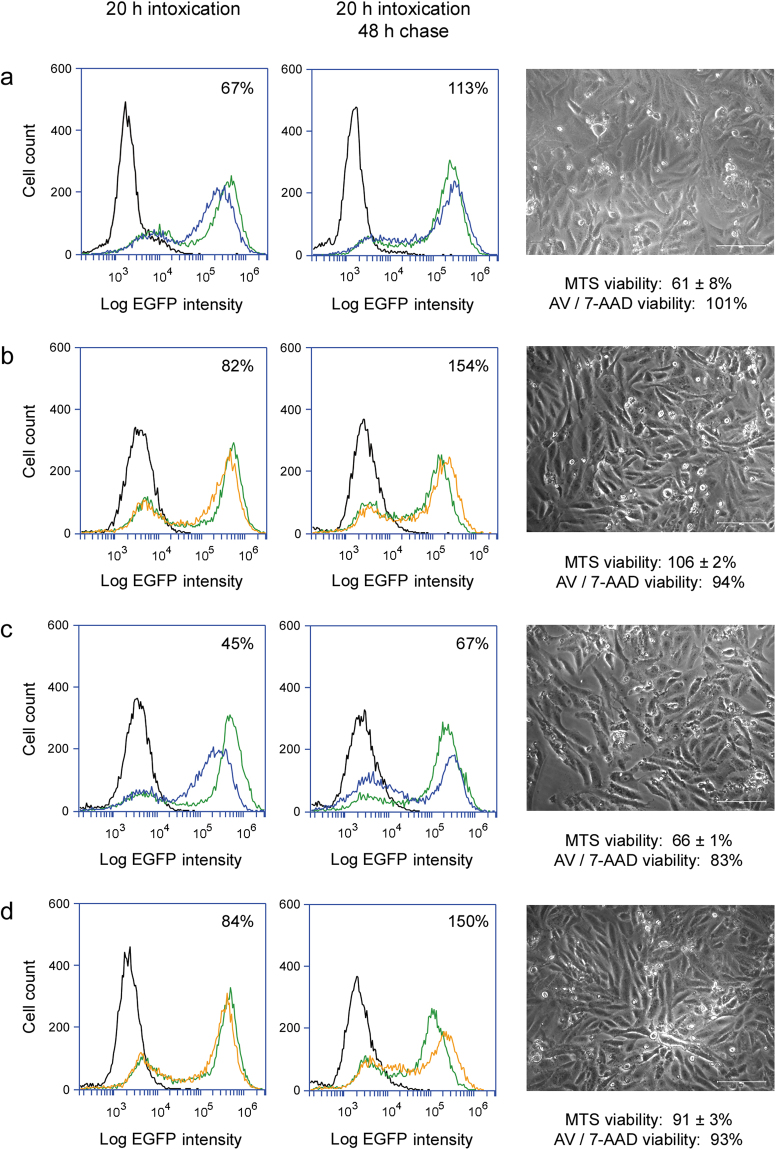
Recovery from intoxication. Vero-d2EGFP cells were incubated with (**a**) 0.025 ng/mL of ricin, (**b**) 0.001 ng/mL of Stx1, (**c**) 1.0 ng/mL of EtxA, or (**d**) 0.01 ng/mL of Dtx for 20 h and then chased for 48 h in the absence of toxin. Blue and orange lines (corresponding to the color-coded toxin concentrations in Supplementary Figure S1) were generated from cytofluorometry analysis of toxin-treated Vero-d2EGFP cells at the end of the 20 h toxin incubation (left column) or 48 h chase (center column). Unintoxicated parental Vero cells (black lines) and unintoxicated Vero-d2EGFP cells (green lines) were also processed for each condition. Percentages represent the strength of the EGFP signal from intoxicated cells in comparison to unintoxicated cells. Right column: Representative images of cells at the end of the 48 h chase were taken at 200× magnification. Cell viability, as assessed by MTS assay (*n* = 3, avg. ± std. dev.) or annexin V (AV) / 7-AAD staining of the same cell population processed for EGFP cytofluorometry, is indicated.

Our collective data suggested cells can tolerate low levels of cytosolic toxin and the partial inhibition of protein synthesis without a terminal effect. Thus, cells could potentially recover from intoxication. To further examine this possibility, we challenged Vero-d2EGFP cells with ricin (Fig. 5a), Stx1 (Fig. 5b), EtxA (Fig. 5c), or Dtx (Fig. 5d) for 20 h. Toxins were applied at concentrations that were at or below the 20 h ED_50_ values. One set of cells for each applied toxin was processed for cytofluorometry, while another set was washed and incubated in toxin-free medium for an additional 48 h before cytofluorometry. The population-wide loss of EGFP fluorescence recorded after 20 h of intoxication demonstrated all cells had a cytosolic pool of toxin at this time, as indicated by the observed downshift in peak fluorescence intensity (Fig. 5, left column). Cells that were chased in toxin-free medium for 48 h exhibited higher levels of fluorescence than were recorded after the initial 20 h toxin challenge (Fig. 5, center column), and a substantial pool of viable, adherent cells remained at the end of the chase (Fig. 5, right column). For these experiments, the quantity of cytosolic toxin present at 20 h of intoxication was therefore insufficient to completely inhibit protein synthesis and kill the entire population of intoxicated cells. Removal of the toxin after a 20 h exposure consequently allowed most cells to recover from the toxin-induced inhibition of protein synthesis.

**Figure 6:**
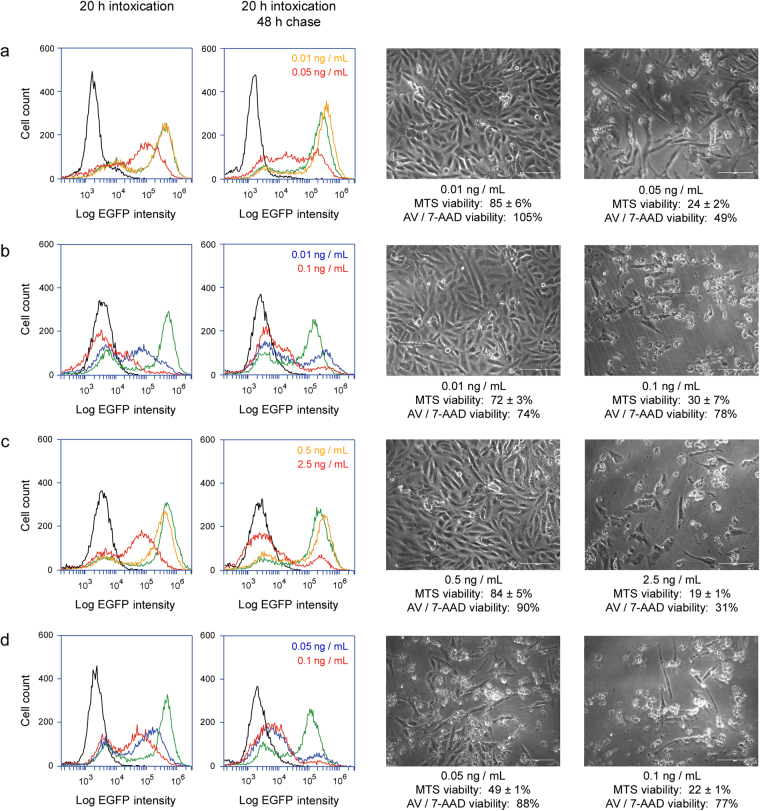
Dose-dependent recovery from intoxication. Vero-d2EGFP cells were incubated with various concentrations of (**a**) ricin, (**b**) Stx1, (**c**) EtxA, or (**d**) Dtx for 20 h and then chased for 48 h in the absence of toxin. Left columns: Orange, blue, and red lines were derived from cytofluorometry analysis of toxin-treated Vero-d2EGFP cells at the end of the 20 h toxin incubation or the end of the 48 h chase. Unintoxicated parental Vero cells (black lines) and unintoxicated Vero-d2EGFP cells (green lines) were also processed for each condition. Right columns: At the end of the 48 h chase, representative images of cells initially exposed to the stated toxin concentrations were taken at 200× magnification. Cell viability, as assessed by MTS assay (*n* = 3, avg. ± std. dev.) or annexin V (AV) / 7-AAD staining of the same cell population processed for EGFP cytofluorometry, is indicated. These experiments were performed at the same time as the data presented in Fig. 5.

As expected, recovery from intoxication was dependent on the applied dose of toxin: cells exposed to higher toxin concentrations did not return to high levels of EGFP fluorescence by the end of the 68 h experiment, and substantial cell death was recorded (Fig. 6). These experiments were performed at the same time as the experiments presented in Fig. 5 and are shown as a separate Figure to emphasize cells can recover from sub-optimal levels of toxin (Fig. 5) but not all toxin concentrations (Fig. 6). Combined, Figs 5 and 6 present data with the three toxin concentrations used throughout this work. Biphasic fluorescent signals were detected for cells exposed to intermediate concentrations of Stx1 or EtxA, which demonstrated our system could detect an all-or-nothing signal distribution with intoxicated cells. It also indicated protein synthesis had been completely inhibited in one subpopulation of toxin-challenged cells, while another subpopulation had completely recovered from intoxication and produced normal levels of protein. The biphasic fluorescent profiles detected in many conditions from the 68 h experiment would skew the mean fluorescent intensities, so d2EGFP signals were not quantified. However, the population-wide loss of protein synthesis recorded at 20 h of intoxication for all toxin concentrations confirmed every cell had at least one molecule of cytosolic toxin at this time. As such, the greater level of cell death resulting from exposure to higher toxin concentrations could not be attributed to a greater number of intoxicated cells (i.e., the probability model of intoxication). Instead, transient exposure to higher toxin concentrations apparently produced a greater quantity of cytosolic toxin that overwhelmed the cellular capacity to withstand intoxication (i.e., the proportionality model of intoxication). Productive intoxication thus requires either transient exposure to high toxin concentrations or continual exposure to sub-optimal toxin concentrations. The exact quantity of toxin required for cell death by either of these mechanisms has yet to be determined, but both scenarios involve exposure to more than one molecule of toxin.

## Discussion

AB toxins are so potent that a single molecule of cytosolic toxin is thought to be sufficient for the complete inhibition of protein synthesis and cell death. The supporting evidence for this model is based upon extrapolations from *in vitro* studies with toxin serial dilutions or kinetic analyses of intoxication^5,11,12^. It would be nearly impossible to directly demonstrate that only one or a few toxin molecules are in the cytosol of a dead cell. As an alternative approach, we have shown cells with a toxin-induced inhibition of protein synthesis can, upon removal of sub-optimal toxin concentrations from the medium, survive the toxin challenge and restore normal levels of protein synthesis. The key experiment involved a 20 h toxin challenge and 48 h recovery period. At least one molecule of toxin must have been present in the cytosol after 20 h of intoxication in order to have an inhibitory effect on protein synthesis, yet the intoxicated cells survived, with normal levels of protein synthesis, 68 h after the initial toxin challenge. We accordingly conclude that one or a few molecules of cytosolic toxin are not sufficient to kill the cell.

Catalytic toxin A chains were originally thought to be stable in the host cytosol^1,15,30,31^, although direct evidence for this assertion is lacking. A single molecule of stable cytosolic toxin could, with time, theoretically block all protein synthesis and kill the cell. However, toxin A chains are not stable in the cytosol: several studies have directly or indirectly documented the proteasome-dependent degradation of cytosolic toxin^5,32–34^. The turnover of cytosolic toxin is apparently faster than the time required for a single toxin molecule to inactivate enough ribosomes for the cessation of protein synthesis and corresponding cell death. Hence, as demonstrated here, productive intoxication requires either ongoing toxin delivery to the cytosol or a large initial bolus of cytosolic toxin.

The “proportionality” model of intoxication suggests the cytosolic stability of the toxin A chain will directly impact the capacity for recovery from intoxication, which is consistent with our observations for Dtx: as shown in Figs 5–6, cells could only recover from a minimal initial inhibition of protein synthesis by this relatively stable toxin^35^. Other reports further suggest the extent of intoxication is linked to the efficiency of toxin clearance from the cytosol. For example, cellular resistance to ricin or EtxA results from enhanced degradation of the cytosolic toxin^33,34,36^. Conditions that impede the turnover of cytosolic toxin likewise generate cellular sensitivity to ricin or Stx1^5,32,33,36^. These collective observations indicate the extent of intoxication is directly linked to how much toxin is in the cytosol, with the amount of cytosolic toxin representing a balance between toxin delivery to the cytosol and toxin removal from the cytosol.

Our study does not minimize the extreme potency of AB toxins, but it does challenge the long-standing assertion that a single or few molecules of cytosolic toxin are sufficient to completely inhibit protein synthesis and kill the cell. Because there is a lag between the inhibition of protein synthesis and cell death, the clearance of cytosolic toxin provides an opportunity to restore normal levels of protein synthesis and recover from transient exposure to sub-optimal toxin concentrations. This was demonstrated here by removing low levels of toxin from the medium. Such observations provide experimental support for the development of inhibitors and post-exposure therapeutics that restrict, but do not necessarily completely block, toxin delivery to the host cytosol. Our observations also indicate the effective application of an anti-cancer immunotoxin^14,15^ will require either ongoing delivery or efficient initial delivery of the toxin A chain into the cytosol of a targeted cancer cell.

## Methods

### Toxins

Ricin was purchased from Vector Laboratories (Burlingame, CA); Stx1 was obtained from BEI Resources (Manassas, VA) or Dr. Alison O’Brien (Uniformed Services University of the Health Sciences); EtxA and Dtx were purchased from List Biologicals (Campbell, CA).

### Fluorescence Measurements

Vero or Vero-d2EGFP cells were seeded in 500 µL volume to black-walled 24 well plates with glass bottom (Cellvis, Mountain View, CA) at a density of 100,000 cells per well. After an overnight incubation at 37 °C with 5% CO_2_, the cells were incubated in serum-free Ham’s F-12 medium containing 20 μg/mL of cycloheximide (Sigma Aldrich, St. Louis, MO) or the stated toxin dilutions. Following incubation, the cells were washed with phosphate-buffered saline (PBS) and then bathed in PBS for EGFP measurement using a Synergy H1 Multi-Mode Microplate Reader (Biotek, Winooski, VT) with bottom optics position and 485 nm excitation / 528 nm emission filter set. For subsequent cytofluorometry analysis, the cells were detached using PBS without calcium and magnesium (GE Healthcare, Logan, UT). EGFP fluorescence was measured using an Accuri C6 Flow Cytometer (BD Biosciences, San Jose, CA). All experiments recorded 10,000 events. For quantification of both plate reader and cytofluorometry data, background levels of autofluorescence from the parental Vero cells were subtracted from the experimental measurements. Background-subtracted data from treated samples were expressed as percentages of the control value obtained from untreated Vero-d2EGFP cells.

### Detection of Apoptosis

Cells treated with toxin in parallel with the fluorescence experiments described above were washed with PBS and detached from the plate with 400 μL PBS lacking calcium and magnesium. The cell suspension was then supplemented with binding buffer containing PE annexin V and 7-AAD (both from BD Biosciences, San Jose, CA). The cell suspensions were protected from light, mixed, and incubated at room temperature for 15 min. Following this incubation, binding buffer was used to bring the final sample volume to 500 µL. Samples were then analyzed using an Accuri C6 Flow cytometer. Unintoxicated cells that were unstained, stained with PE annexin V alone, or stained with 7-AAD alone were used to establish the quandrant for healthy, viable cells lacking annexin V and 7-ADD staining. The fraction of unintoxicated Vero-d2EGFP cells in this quandrant was arbitrarily set as the 100% control value, and the fraction of viable cells after toxin or H_2_O_2_ challenge were expressed as percentages of the control value.

### MTS Viability Assay

To monitor cell viability through cellular metabolism, 20,000 Vero-d2EGFP cells were seeded in a 96-well plate and allowed to reach ~80% confluency overnight at 37 °C under 5% CO_2_. Cells were then treated with cycloheximide, H_2_O_2_, or toxins diluted in serum-free Ham’s F-12 medium. Following the indicated incubation period, 20 µL of MTS reagent (Promega, Madison, WI) was added to each well of the plate and incubated for 3 h at 37 °C. NADPH and NADH from live, metabolically active cells reduce the MTS reagent into a colored formazan product that can be detected at an absorbance of 490 nm using a Synergy H1 Multi-Mode Microplate Reader. Absorbance is directly proportional to the extent of cell viability. Background readings taken from wells without cells were subtracted from the experimental measurements. After background subtraction, the absorbance value obtained from untreated control cells was arbitrarily set at 100%. Data from treated or intoxicated samples were then expressed as percentages of the control value. Each experiment was run with 6 to 12 replicate samples per condition.

### Light Microscopy

Vero-d2EGFP cells were seeded to 24 well plates in 500 uL volume at a density of 100,000 cells per well. After growth overnight at 37 °C and 5% CO_2_, the cells were treated with toxins diluted in serum-free Ham’s F-12 medium. Following incubations of the indicated times, phase contrast pictures were taken using a Nikon Eclipse TE200 microscope equipped with 20 × objective lens and a Nikon Digital Sight camera (Nikon Instruments Inc., Melville, NY). The intoxicated cells were not washed prior to image capture.

### Data Availability

The datasets generated during and/or analyzed during the current study are available from the corresponding author on reasonable request.

## Electronic supplementary material

Supplementary Data
