# Supplementary material for: Cellular recovery from exposure to sub-optimal concentrations of AB toxins that inhibit protein synthesis
Source: Sci Rep. 2018 Feb 6;8:2494. doi: 10.1038/s41598-018-20861-9 (PMC5802730; doi:10.1038/s41598-018-20861-9)
Supplement: Supplementary file 1 — Supplementary Data [file 41598_2018_20861_MOESM1_ESM.doc]

Cellular recovery from exposure to sub-optimal concentrations of AB toxins that inhibit protein synthesis

Patrick Cherubin, Beatriz Quiñones, and Ken Teter

Supplementary Data


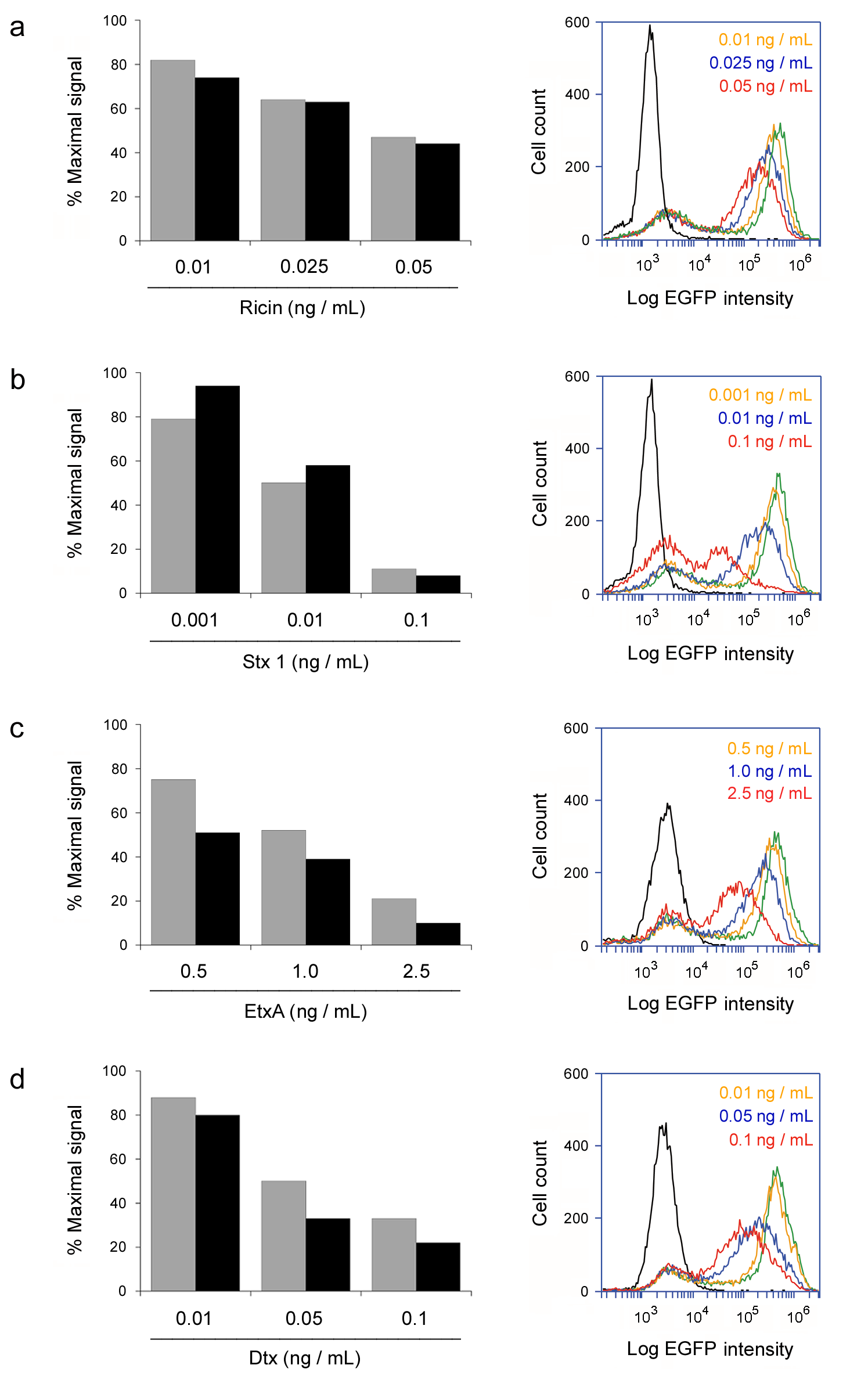


**Supplementary Figure S1.** Dose-dependent, population-wide loss of fluorescence from toxin-treated Vero-d2EGFP cells. Cells were incubated for 20 h with various concentrations of (**a**) ricin, (**b**) Stx1, (**c**) EtxA, or (**d**) Dtx. Left column: Using data collected from the same cells by either cytofluorometry (grey bars) or with a plate reader (black bars), signals from the toxin-treated cells were expressed as percentages of the value recorded for untreated Vero-d2EGFP cells. Right column: Cytofluorometry data from the same pool of intoxicated cells analyzed in the left column are shown, along with results from unintoxicated parental Vero cells (black lines) and unintoxicated Vero-d2EGFP cells (green lines). Orange, blue, and red lines were generated from Vero-d2EGFP cells incubated with the color-coded toxin concentration. These experiments were performed at the same time as the data presented in Figure 3 and, for comparative purposes, include the toxin concentrations used in Figure 3.


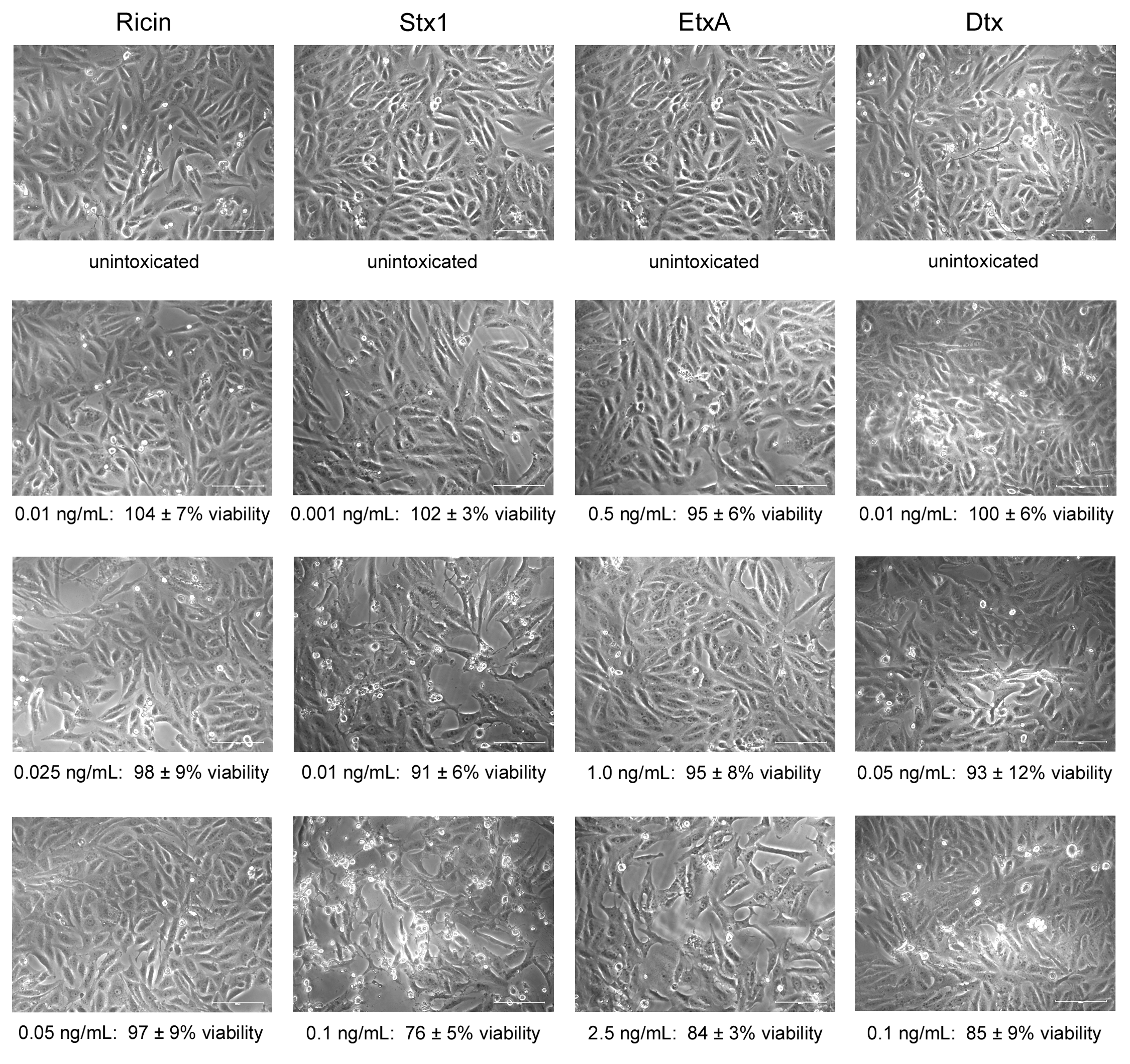


**Supplementary Figure S2.** Cell viability after 20 h intoxications. As indicated, Vero-d2EGFP cells were incubated for 20 h with various concentrations of ricin, Stx1, EtxA, or Dtx. Representative images were taken at 200× magnification. Viability, as assessed by MTS assay (*n* = 3, avg. ± std. dev.), is reported below each image along with the applied dose of toxin.


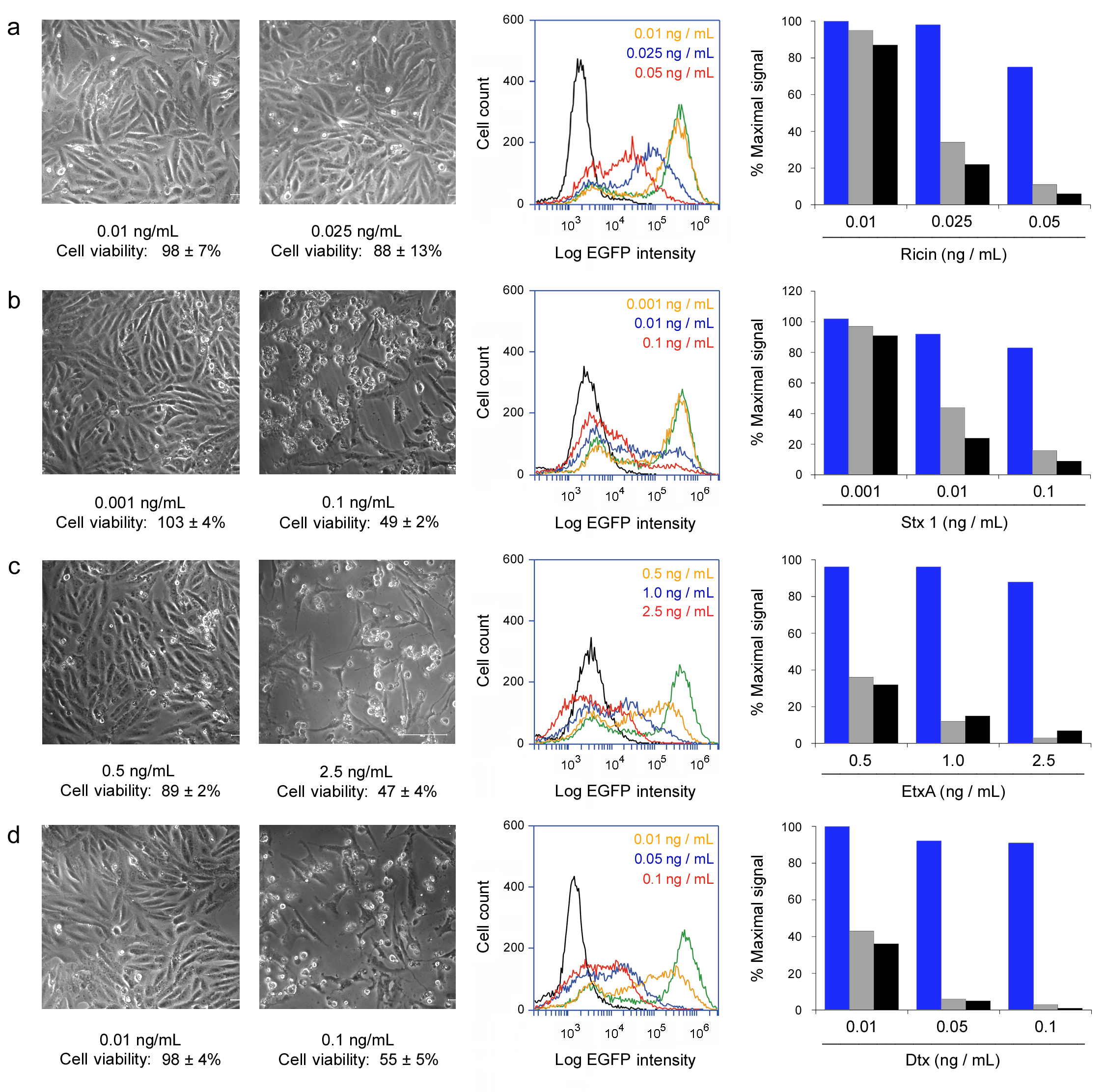


**Supplementary Figure S3.** Effect of long-term toxin exposure on cell viability and protein synthesis. Vero-d2EGFP cells were incubated for 36 h with various concentrations of (**a**) ricin, (**b**) Stx1, (**c**) EtxA, or (**d**) Dtx. Left columns: Representative images were taken at 200× magnification. Cell viability, as assessed by MTS assay (*n* = 3, avg. ± std. dev.), is indicated along with the applied dose of toxin. Center column: The subpopulation of adherent cells were subjected to cytofluorometry, along with unintoxicated parental Vero cells (black lines) and unintoxicated Vero-d2EGFP cells (green lines). Orange, blue, and red lines were derived from Vero-d2EGFP cells incubated with the color-coded toxin concentration. Right column: Cell viability was recorded by cytofluorometry analysis of annexin V and 7-AAD staining (blue), while EGFP fluorescence was recorded by cytofluorometry (grey) or with a plate reader (black). Results are presented as percentages of the values obtained from unintoxicated cells. All measurements in the matched center and right columns were performed on the same population of cells. These experiments were performed at the same time as the data presented in Figure 4 and, for comparative purposes, include the toxin concentrations used in Figure 4.
